# Closing Editorial: Colorectal Cancer—A Molecular Genetics Perspective

**DOI:** 10.3390/ijms252312604

**Published:** 2024-11-24

**Authors:** Ehsan Gharib

**Affiliations:** 1 Département de Chimie et Biochimie, Université de Moncton, Moncton, NB E1A 3E9, Canada; ehsangharib55@gmail.com; 2 Atlantic Cancer Research Institute, Moncton, NB E1C 8X3, Canada

Colorectal cancer (CRC) remains a significant global health challenge, ranking third in incidence and second in mortality among all cancers [1]. Despite advancements in screening, diagnosis, and treatment, the rising prevalence of early-onset colorectal cancer (EOCRC) in individuals under 50 years old is particularly concerning [2,3]. This alarming trend highlights the need for a better understanding of the underlying molecular mechanisms and risk factors associated with EOCRC [4]. Furthermore, the development of more effective screening strategies and targeted interventions for this younger patient population is crucial to improve outcomes and reduce the burden of CRC [5,6,7,8,9]. This Special Issue, titled “Colorectal Cancer: A Molecular Genetics Perspective”, showcases the latest research and developments in understanding the molecular mechanisms underlying CRC and their potential applications in improving patient outcomes. The studies presented here provide valuable insights into the complex genetic and epigenetic landscape of CRC, paving the way for novel diagnostic, prognostic, and therapeutic approaches.

The articles in this Special Issue cover a wide range of topics, varying from the construction of multi-gene risk models for CRC prognosis and drug treatment prediction [10] to the identification of novel circulating mRNA [11] and miRNA biomarkers for non-invasive diagnosis [12]. These studies underscore the potential of leveraging molecular profiling techniques to develop more precise and personalized risk assessment tools and early detection methods. The role of specific genetic alterations, such as KRAS mutations, in CRC-associated thrombosis [13] and therapeutic resistance [14] is also explored, highlighting the importance of understanding the functional consequences of these genetic changes in the context of CRC pathogenesis and treatment. Additionally, the importance of epigenetic regulation, including DNA methylation, histone modifications, and non-coding RNAs, in CRC pathogenesis and their potential as diagnostic and prognostic markers are highlighted [15]. These findings emphasize the need to integrate epigenetic data with genetic information to gain a more comprehensive understanding of CRC biology and identify novel therapeutic targets.

Several studies in this Special Issue focus on the molecular heterogeneity of CRC and its implications for personalized medicine. The consensus molecular subtypes (CMS1-CMS4) are examined, shedding light on disease heterogeneity and their potential for guiding targeted therapies [16]. These subtypes, based on gene expression profiles, provide a framework for stratifying CRC patients and tailoring treatment strategies based on their molecular characteristics [16,17]. The genetic alterations in the NF-κB signaling pathway, estimated to account for over 50% of the overall genetic changes in CRC patients, are also discussed as promising targets for novel therapeutic development [18]. Given the central role of NF-κB signaling in various aspects of CRC progression, including inflammation, cell survival, and metastasis, targeting this pathway could potentially lead to more effective and specific therapies for CRC patients harboring these alterations [18,19,20].

While this Special Issue contributes significantly to our understanding of the molecular genetics of CRC, gaps in knowledge remain. For example, the variability in the diagnostic performances of circulating miRNA biomarkers underscores the need for standardized methods and robust validation studies in large-scale, ethnically diverse cohorts [12,21]. This is particularly important given the potential of these biomarkers for non-invasive, early detection of CRC, which could greatly improve patient outcomes [12,21]. Furthermore, the molecular mechanisms underlying EOCRC and its rising incidence require further investigation [4,22,23]. Identifying the unique genetic, epigenetic, and environmental risk factors associated with EOCRC will be crucial for developing targeted prevention and screening strategies for this growing subset of patients [4,22,23].

To address these gaps and advance the field, future research should focus on several key areas. First, the development of more representative and diverse CRC models, such as patient-derived xenografts (PDX) [24], organoids [25], and isogenic human embryonic stem cell (hESC) or human pluripotent stem cell (hPSC)-derived colonic cells [26], will be crucial for uncovering the underpinning mechanisms of genetic alterations in CRC progression. These models provide a more physiologically relevant context for studying CRC biology and testing novel therapeutic approaches, as they better recapitulate the complexity and heterogeneity of human tumors compared to traditional cell line models [24,25,26]. Second, establishing platforms for drug discovery [18,27] and biobanks [28,29] will facilitate the development of innovative personalized medicine approaches for CRC treatment. By leveraging high-throughput screening technologies and large-scale biospecimen collections, researchers can identify novel drug targets [30] and biomarkers [31] and develop more precise and effective therapies tailored to individual patient characteristics [32]. Finally, continued efforts to elucidate the molecular basis of EOCRC and identify risk factors and preventive strategies for this growing subset of patients are essential [33,34]. This will require a multidisciplinary approach, integrating epidemiological, clinical, and molecular data to gain a more comprehensive understanding of EOCRC etiology and develop evidence-based interventions [5].

In addition to the topics covered in this Special Issue, there are several other areas of CRC research that warrant further exploration. One such area is the role of the gut microbiome in CRC development and progression [35,36,37]. Emerging evidence suggests that alterations in the gut microbiome composition and function may contribute to CRC pathogenesis [35,36] and modulating the microbiome could potentially serve as a preventive or therapeutic strategy [37]. Studies have shown that certain bacterial species, such as Fusobacterium nucleatum [38] and Bacteroides fragilis [39], are enriched in CRC tumors and may promote tumorigenesis through various mechanisms, including inflammation, immune evasion, and metabolic reprogramming. Investigating the complex interactions between the gut microbiome, host genetics, and environmental factors in the context of CRC could lead to novel microbiome-based diagnostic, prognostic, and therapeutic approaches [40,41,42]. Another promising avenue of research is the application of artificial intelligence and machine learning techniques to analyze large-scale genomic [42], transcriptomic [43], and proteomic data from CRC patients [44]. These approaches could help to identify novel biomarkers, predict treatment responses, and guide personalized therapy decisions [42,43,44]. By integrating multiple layers of molecular data and clinical information, AI-based models could potentially improve the accuracy and efficiency of CRC risk assessment, diagnosis, and treatment planning [42,43,44].

Moreover, the field of immunotherapy has shown great promise in the treatment of various cancers, including CRC [45]. Investigating the immune landscape of CRC tumors and developing strategies to enhance the efficacy of immune checkpoint inhibitors [46] and adoptive cell therapies [47] could lead to improved outcomes for patients with advanced or metastatic disease. Recent studies have identified several immune-related biomarkers, such as microsatellite instability (MSI) status [48] and tumor mutational burden (TMB) [49], that predict response to immunotherapy in CRC patients. Further research into the molecular mechanisms underlying immune evasion and resistance in CRC, as well as the development of novel immunotherapeutic approaches, such as personalized neoantigen vaccines [50] and combination therapies [45], could expand the benefits of immunotherapy to cover a larger proportion of CRC patients. Additionally, exploring the potential of combination therapies that target multiple molecular pathways or combine conventional chemotherapy with targeted agents or immunotherapies may help to overcome therapeutic resistance and improve patient survival [51,52,53,54]. As our understanding of the complex signaling networks and feedback loops involved in CRC progression grows, designing rational combination strategies that exploit synergistic effects and minimize toxicities will become increasingly important.

In conclusion, this Special Issue provides a comprehensive overview of the current state of molecular genetics research in CRC, highlighting recent advancements, addressing knowledge gaps, and setting the stage for future breakthroughs. The studies presented here demonstrate the power of integrating molecular profiling, functional genomics, and translational research approaches to unravel the complexities of CRC biology and develop more precise and effective diagnostic, prognostic, and therapeutic strategies. As our understanding of the complex interplay between genetic, epigenetic, and environmental factors in CRC pathogenesis continues to evolve, we can anticipate the development of more precise diagnostic tools, prognostic markers, and targeted therapies that will ultimately improve outcomes for patients with this prevalent and deadly disease. However, significant challenges remain, particularly in the areas of EOCRC, molecular heterogeneity, therapeutic resistance, and immune evasion. Addressing these challenges will require a sustained and collaborative effort from the global research community, involving multidisciplinary teams of basic scientists, clinicians, bioinformaticians, and patient advocates. Moving forward, a multidisciplinary approach that integrates expertise from basic science, translational research, clinical practice, and bioinformatics will be essential to tackle the remaining challenges in CRC prevention, early detection, and personalized treatment. By fostering cross-disciplinary collaborations, sharing data and resources, and prioritizing patient-centered research, we can accelerate the pace of discovery and translation, ultimately leading to a future where CRC is a preventable, treatable, and potentially curable disease for all patients, regardless of age or molecular subtype.
